# Rescue stenting after the failure of mechanical thrombectomy to treat acute intracranial atherosclerotic occlusion

**DOI:** 10.3389/fneur.2022.1001496

**Published:** 2023-01-10

**Authors:** Shunyuan Guo, Tianyu Jin, Chao Xu, Wei Huang, Zongjie Shi, Yu Geng

**Affiliations:** ^1^Department of Clinical Medicine, Medical College of Soochow University, Suzhou, Jiangsu, China; ^2^Center for Rehabilitation Medicine, Department of Neurology, Zhejiang Provincial People's Hospital, Affiliated People's Hospital, Hangzhou Medical College, Hangzhou, Zhejiang, China; ^3^Department of Neurology, Zhejiang Chinese Medical University, Hangzhou, China; ^4^Rheumatism and Immunity Research Institute, Zhejiang Provincial People's Hospital, Affiliated People's Hospital, Hangzhou Medical College, Hangzhou, China

**Keywords:** rescue stenting, mechanical thrombectomy, symptomatic intracerebral hemorrhage, acute ischemic stroke, intracranial atherosclerosis

## Abstract

**Background:**

Acute ischemic stroke (AIS) with intracranial large vessel occlusion (LVO) is refractory to reperfusion because of the underlying intracranial atherosclerosis (ICAS), and this condition often requires salvage methods such as balloon angioplasty and rescue stenting (RS). In this study, we investigated the short-term outcomes of RS after failed mechanical thrombectomy (MT) for the treatment of acute intracranial atherosclerotic occlusion.

**Methods:**

We retrospectively evaluated the clinical data of 127 patients who underwent MT for acute intracranial atherosclerotic occlusion in our hospital between August 2018 and January 2022. The degree of recanalization was evaluated immediately after the treatment by Modified Thrombolysis in Cerebral Infarction (mTICI). The modified Rankin Scale (mRS) was used 90 days after treatment to evaluate the neurological functions. In addition, the incidence of symptomatic intracranial hemorrhage (sICH) and postoperative mortality within 90 days of treatment were calculated.

**Results:**

Among the 127 patients, 86 patients (67.7%) had revascularization (mTICI 2b-3) immediately after MT (non-RS group), and RS was performed in 41 patients (32.3%) after MT failure (RS group). No difference in the sICH rate was observed between the two groups (17.1 vs. 16.3%, *p* = 0.91). There was a slightly higher mortality rate in the RS group (14.6 vs. 12.8%, *p* = 0.71); however, the difference was not significant. There was no difference in the proportion of patients in the RS and non-RS groups who had a 90-day mRS score of 0–2 (48.8 vs. 52.3%, *p* = 0.76).

**Conclusions:**

Rescue stenting after MT failure might be a feasible rescue modality for treating acute intracranial atherosclerotic occlusion.

## Introduction

Acute ischemic stroke (AIS) with intracranial large vessel occlusion (LVO) is an emergency medical condition that is associated with high disability and mortality rates. Mechanical thrombectomy (MT) has been recommended as a first-line method for treating AIS with LVO (1). However, MT fails to achieve successful recanalization (modified thrombolysis in cerebral ischemia [mTICI], 2b-3) in 20–30% of patients, even with the newest generation of mechanical devices (2, 3). There are several causes of MT failure. One of the major causes is atherosclerosis-related *in situ* stenosis or occlusion. In particular, intracranial atherosclerotic stenosis (ICAS) is more common in Chinese populations and other Asian populations than in Western populations, and in these populations, the recanalization failure rate of MT may be higher (4, 5); thus, rescue measures are needed to maintain vessel patency in these refractory patients. In recent years, rescue stenting (RS) has been considered to be the most promising salvage method for patients for whom MT fails (6, 7). A multicenter prospective study found that RS after failed MT increased the sICH and mortality (5.1 vs. 4.0%) compared with the patients without RS (8). Moreover, RS may at a risk for acute stent thrombosis and in-stent restenosis (9.6%) (9); additionally, its safety and efficacy are not yet clear, and its clinical application is controversial. We retrospectively evaluated the clinical data of 127 patients who underwent MT for acute intracranial atherosclerotic occlusion in our hospital between August 2018 and January 2022 in order to investigate the short-term outcomes of RS after the failure of MT in patients with acute intracranial atherosclerotic occlusion.

## Methods

### Ethics statement

The human ethics committee of the Zhejiang Provincial People's Hospital approved the protocol of this study. All the clinical investigations were conducted according to the principles outlined in the Declaration of Helsinki. All subjects provided written informed consent before the study.

### Patients

All the included patients received endovascular treatment between August 2018 and January 2022 in the stroke medical center of Zhejiang Provincial People's Hospital. Stent placement was used as a rescue therapy after MT failure in patients with AIS because of intracranial atherosclerotic occlusion.

Patients were included in this study if (1) they were aged ≥18 years; (2) they had an onset-to-puncture time ≤24 h; (3) they had a modified Rankin Scale (mRS) score of <2 before stroke; (4) they had AIS caused by ICAS-LVO, which was diagnosed by 70% of residual stenosis after first-pass thrombectomy (10, 11); and (5) they or their legal guardian signed the informed consent form for the operation. This retrospective study was approved by the ethics committee of the involved centers.

### Intravenous thrombolysis

Patients who were eligible for intravenous thrombolysis (IVT, recombinant tissue plasminogen activator 0.9 mg/kg) received IVT before endovascular treatment. Patients were administered 10% of the total dose in the first bolus, and the remaining 90% of the dose was intravenously administered within 1 h of the initial bolus.

### Endovascular therapy

**Figure 1:**
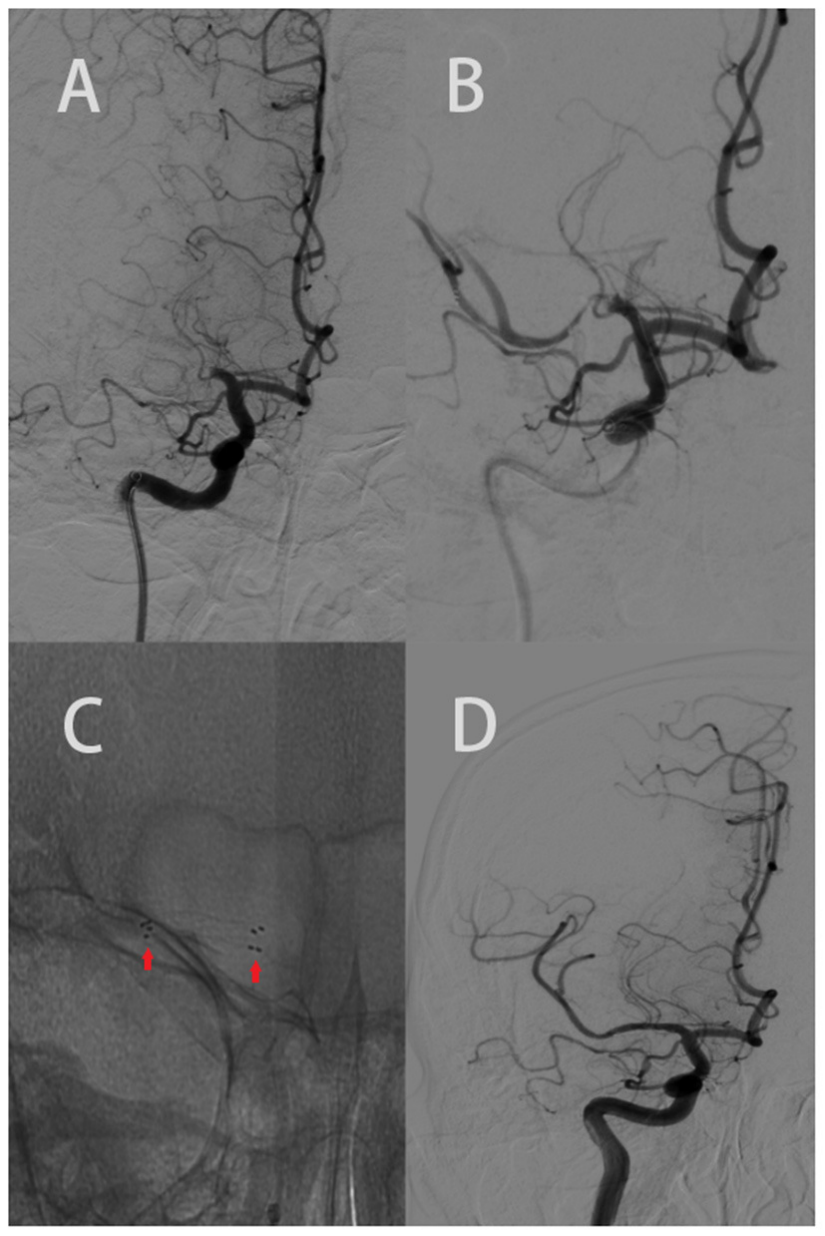
Elderly patient with an acute onset of left body weakness, numbness, and difficulty speaking (NIHSS 7). The ASPECTS was 9 based on CT scan. **(A)** AP right carotid angiogram clearly showed an occlusion of the M1 segment of the right MCA. **(B)** Revealed the post failed MT with stenosis. **(C)** The red arrow shows the proximal and distal legs of the stent. **(D)** AP right carotid angiogram depicting recanalization of the right MCA and its branches (mTICI 3).

Endovascular therapy (EVT) was performed after successful femoral artery catheterization under general anesthesia or conscious sedation. The type of MT device that was used was a Solitaire AB. RS was performed as a salvage measure in cases of failed MT with identified residual stenosis >70% during MT (>70% stenosis according to the Warfarin Aspirin Symptomatic Intracranial Disease criteria). If necessary, high-pressure balloon angioplasty was performed before and after RS placement (Figure 1). The inclusion criteria were as follows: (1) identified residual ICAS >70% with reocclusion after MT and (2) insufficient distal flow restoration despite recanalization after MT. Figure 1 shows the process of the RS implantation.

### Perioperative antithrombotic therapy

**Figure 2:**
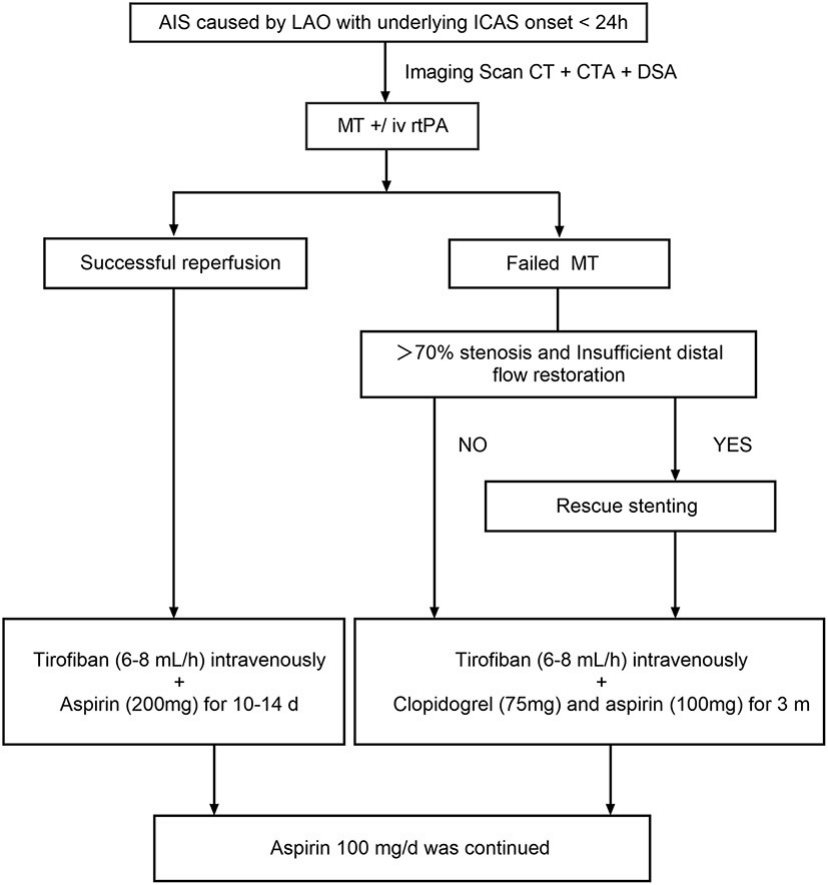
Flow chart of the MT process in out center. AIS, acute ischemic stroke; LVO, intracranial large vessel occlusion; CT, computed tomography; CTA, computed tomographic angiography; DSA, digital subtraction angiography; MT, mechanical thrombectomy; rtPA, recombinant tissue plasminogen activator. MT +/ iv rt PA, iv rtPA is performed if the patient is within 4.5 h of onset.

The procedure was performed with systemic heparin administration to maintain the activated clotting time of 250–300 s. Tirofiban (6–8 mL/h) was continuously administered intravenously within 24 h after rescue stent deployment. If there was no bleeding upon re-examination of cranial CT, dual antiplatelet drug therapy with clopidogrel (75 mg) and aspirin (100 mg) was overlapped with one-half the dose of the intravenous tirofiban infusion 4 h before its cessation; this was followed by the administration of 75 mg/days clopidogrel and 100 mg/days aspirin for 3 months. Clopidogrel treatment was stopped after 3 months, and 100 mg/days aspirin was continued. In the non-RS group, aspirin (200 mg) was used for 10–14 days rather than dual antiplatelet drug therapy. Patients were recommended to continue taking aspirin for life; once an intracranial hemorrhage is found, antiplatelet therapy should be stopped. The outline of the MT process in our center is shown in Figure 2.

### Clinical and imaging follow-up

The National Institutes of Health Stroke Scale (NIHSS) score at admission was used to evaluate the severity of stroke. All the patients were examined *via* MR imaging or CT in the first 24 h following EVT or when neurological deterioration occurred. mTICI was used to evaluate reperfusion status; successful reperfusion was defined as mTICI ≥ 2b/3. SICH was defined as the identification of any type of intracranial hemorrhage by imaging studies and an increase in the NIHSS score to ≥4 from any baseline. Parenchymal hematoma type 1 (PH1) represented a blood clot not exceeding 30% of the infarcted area with mild space-occupying effect. Parenchymal hematoma type 2 (PH-2) was defined as dense blood clot(s) exceeding 30% of the infarcted area with significant space-occupying effect (12). Clinical outcomes were assessed *via* the modified Rankin Scale (mRS) at 90 days, and this evaluation was conducted by the telephone or during outpatient visits; a good outcome was defined as a mRS of 0–2.

### Statistical analysis

All the data were analyzed by SPSS software (version 22.0; IBM Corp., Armonk, New York, USA). Categorical variables are expressed as a number. Continuous variables are presented as the median ± interquartile range or mean ± standard deviation (SD). Baseline characteristics and clinical outcomes of the two groups were compared with the χ^2^ test, Student's *t*-test, and rank sum test for continuous variables. Reported probability values were two-sided. A value of *p* < 0.05 was considered significant.

## Results

**Figure 3:**
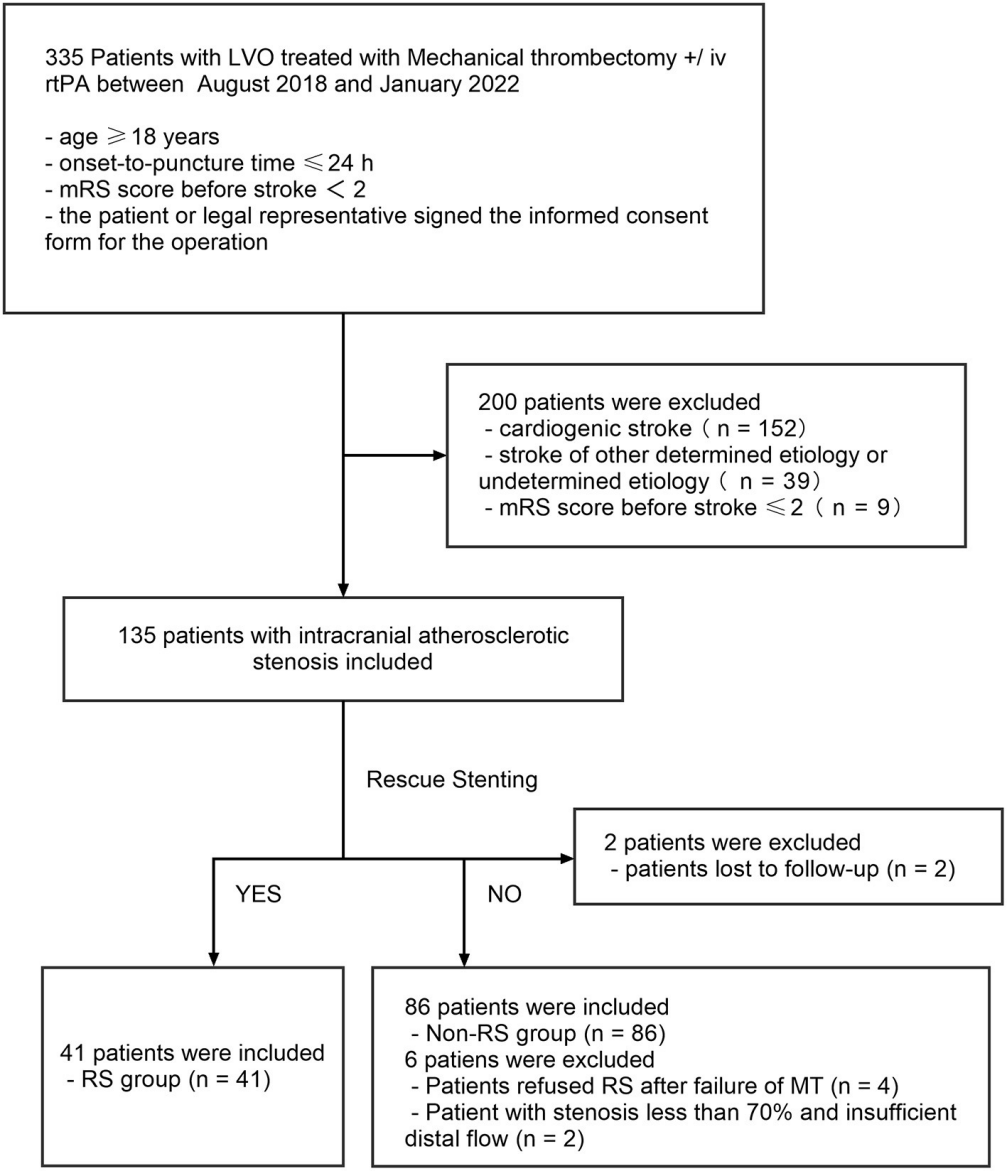
Flow chart of patient inclusion in the study. LVO, intracranial large vessel occlusion; RS, rescue stenting.

The outline of the patient selection process is demonstrated in Figure 3. A total of 335 patients at our center received MT for AIS from August 2018 to January 2022. Of these patients, 200 were excluded for cardiogenic stroke (*n* = 152), stroke of other determined etiology or undetermined etiology (*n* = 39), and mRS score before stroke ≥ 2 (*n* = 9). Therefore, 135 patients with acute intracranial atherosclerotic occlusion were included in the study. In total, eighty-six patients (63.7%) achieved revascularization (mTICI 2b/3) immediately after mechanical thrombectomy (non-RS group). Overall, forty-one patients (30.3%) underwent stenting placement after the failure of mechanical thrombectomy (RS group), including 29 patients (70.7%) with anterior circulation and 12 patients (29.3%) with posterior circulation. Moreover, eight patients (5.9%) were excluded due to refused RS (*n* = 4), stenosis <70% and insufficient distal flow (*n* = 2), and lost to follow-up (*n* = 2). Of the 127 patients finally included in RS group and non-RS group, a total of 54 patients have more than 70% ICAD residual stenosis by WASID. All the included patients eventually achieved successful revascularization (mTICI ≥ 2b/3).

No significant differences were found in the baseline characteristics between the RS group and non-RS group. Patients in the RS group had a higher number of MT passes (2 [IQR, 2–2.5] vs. 1 [IQR, 1–2], *p* = 0.00) and had longer procedural times (64 [IQR, 48–75] vs. 52 [38–69.5] min, *p* = 0.01) (Table 1).

**Table 1:** Comparison of baseline demographics and clinical relevance characteristics among two groups.

**Clinical characteristics**	**RS group** **(*n* = 41)**	**Non-RS group** **(*n* = 86)**	***H*/*t*/χ^2^/*Z***	***P-*value**
Age, (year; mean ± SD)	61.98 ± 13.70	63.16 ± 13.39	−0.46	0.64
Male sex, *n* (%)	31 (75.6%)	63 (73.3%)	0.08	0.78
**Risk factors**, ***n*** **(%)**				
Hypertension	29 (70.7%)	64 (74.4%)	0.19	0.66
Diabetes mellitus	10 (24.4%)	22 (25.6%)	0.02	0.89
Dyslipidemia	21 (51.2%)	40 (46.5%)	0.25	0.62
Coronary artery disease	1 (2.4%)	4 (4.7%)	0.55	1.00
Smoking	13 (31.7%)	29 (33.7%)	0.82	1.00
**Parameters on admission**				
Platelets (G/L; median, IQR)	182 (135.50, 238)	193.5 (158.75, 233.50)	−0.95	0.34
Glucose (mmol/L; median, IQR)	7.21 (6.57, 9.12)	7.19 (6.11, 9.09)	−0.20	0.84
Systolic BP (mmHg; mean ± SD)	163.48 ± 22.70	154.45 ± 26.46	1.36	0.25
Baseline NIHSS score (median, IQR)	15 (11, 21.5)	16 (12, 23)	−0.43	0.67
Onset to admission time (mins; median, IQR)	360 (202.5, 692)	349 (180.5, 675)	−0.57	0.57
ASPECT (median, IQR)	9 (8, 9.5)	9 (8, 9)	−0.15	0.88
**Occlusion sites**, ***n*** **(%)**				
Internal carotid artery	11 (26.9%)	24 (27.9%)	0.02	0.90
Middle cerebral artery	18 (43.9%)	47 (54.6%)	1.28	0.26
Basilar artery	6 (14.6%)	6 (7.0%)	0.20	0.15
Vertebral artery	6 (14.6%)	9 (10.5%)	0.46	0.50
Intravenous thrombolysis, *n* (%)	18 (43.9%)	29 (34.1%)	1.13	0.29
General anesthesia [*n* (%)]	39 (95.1%)	76 (88.4%)	1.48	0.22
Onset to puncture time (mins; median, IQR)	489 (319.5, 847.5)	513 (325, 824.5)	−0.39	0.70
Procedure duration (mins; median, IQR)	64 (48, 75)	52 (38, 69.5)	2.60	0.01
Passes of retriever, median (IQR)	2 (2, 2.5)	1 (1, 2)	−4.47	0.00

NIHSS, national institutes of health stroke scale; SD, standard deviation; IQR, inter quartile range; BP, blood pressure; ASPECT, Alberta stroke program early CT score; RS, rescue stenting.

There were no patients who suffered acute in-stent thrombosis (within 24 h of initial placement) or subacute in-stent thrombosis (24 h to 1 month of initial placement) (13), and sICH occurred in 7 patients (17.1%) in the RS group. Following sICH, two patients developed massive bleeding due to artery perforation during the stent placement procedure and died 3 days after the operation, three patients developed bleeding within 24 h after surgery, and two patients developed bleeding 2 days after surgery. In total, twenty patients (48.8%) achieved a favorable outcome (mRS = 0–2), and six patients (14.6%) died before the 90-day follow-up. The favorable outcome rates at 90 days, mortality rates, sICH rates, and PH rates were comparable between the RS group and the non-RS groups (Table 2).

**Table 2:** Comparison of clinical outcomes among the two groups.

**Clinical outcomes**	**RS group** **(*n* = 41)**	**Non-RS group** **(*n* = 86)**	**χ^2^**	***P-*value**
sICH [*n* (%)]	7 (17.1%)	14 (16.3%)	0.01	0.91
PH [*n* (%)]	5 (12.2%)	9 (10.5%)	0.09	0.77
PH-1	2 (4.9%)	3 (3.5%)	0.14	0.71
PH-2	3 (7.3%)	6 (7.0%)	0.01	0.94
mRS ≤ 2 at 90 days [*n* (%)]	20 (48.8%)	45 (52.3%)	0.14	0.71
Mortality [*n* (%)]	6 (14.6%)	11 (12.8%)	0.08	0.76

sICH, symptomatic intracranial hemorrhage; PH, parenchymal hematoma; mRS, modified ranking scale; RS, rescue stenting.

## Discussion

Mechanical thrombectomy significantly improved the rates of recanalization and the prognosis of patients with AIS with LVO (1). However, a meta-analysis of five clinical studies on MT found that recanalization (mTICI = 0–2a) was not successful in 29% of patients with AIS who were treated with MT (2). Even with the continued improvement of instruments, technologies, and methods, the failure rate of MT is still as high as 20% (3). There are a few causes of MT failure. ICAS *in situ* thromboocclusion is undoubtedly one of the most common causes of AIS with LVO (6, 7, 14, 15), especially in Asian populations; in fact, it may account for one-third of intracranial LVO in Asian populations (4). AIS with LVO related to ICAS is an important challenge for emergency EVT. In ICAS with LVO, even if successful recanalization is initially realized by MT, it is easy for reocclusion to result in MT failure due to the failure to relieve *in situ* vascular stenosis and stent injury to the vascular endothelium (16). The natural history of non-recanalized AIS with LVO is poor (17). Similarly, the outcome of patients who experience MT failure is also poor (18), and remedial measures are needed to maintain vascular patency. However, the optimal treatment for ICAS underlying LVO in patients with AIS remains unclear. Recently, several approaches have been used to try to maintain blood flow for ICAS underlying LVO, such as glycoprotein IIb/IIIa inhibitors, including tirofiban, balloon angioplasty, and stenting (19); among these approaches, RS is considered to be the most promising rescue approach to maintain vessel patency in patients with repeated reocclusion (6, 7, 14, 15). In the case of unsuccessful MT, RS may be the only treatment option to achieve permanent recanalization and further functional independence. A meta-analysis that included 12 studies showed that RS after failed MT significantly improved clinical outcomes (48.5 vs. 19.7%, *p* < 0.01) and did not increase the rate of sICH (9.7 vs. 14.1%, *p* = 0.04) compared with the patients without RS (9). As a remedial treatment after MT failure, RS is characterized by high safety and effectiveness; the results of previous studies have indicated that the rate of successful recanalization (mTICI ≥ 2b/3) after RS is 64.6–98.2%, good functional 90-day postoperative clinical outcomes (mRS = 0–2) are observed in 34.6–66% of patients, and favorable outcomes of RS are equivalent to the results of successful recanalization by MT (20–28). In this study, the rate of favorable outcomes in the RS group at 90 days was 48.8% (20/41), and this was comparable to that in the non-RS group (52.3%). Therefore, RS after failed MT is an effective method for acute intracranial atherosclerotic occlusion.

sICH is part of the natural course of AIS; however, it is also the most important and serious complication of reperfusion treatment and is related to poor prognosis (29) also in addition to being the main cause of insufficient thrombolysis and EVT (30). Controlling the risk of bleeding plays an important role in the decision to attempt recanalization in AIS. After MT, the risk of HT may increase due to the recovery of blood flow. A meta-analysis of five studies on MT found that the incidence of sICH after MT was 4.4% (2). In two trials of MT extended treatment time windows, the incidence of sICH in the treatment group was 6–7% (31, 32). However, in real-world studies (33), the sICH rate is higher than that in randomized trials (2, 31, 32). Permanent stent implantation in AIS may further increase the risk of HT by making additional antiplatelet therapy necessary to prevent stent thrombosis. Studies have shown that the incidence of sICH in RS is 0–16.7%, the PH rate is 0–16.1%, and the mortality rate is 0–31.9% (20–28). In this study, the PH rate was 12.2% (5/41), and the mortality rate was 14.6% (6/41), which were similar to the previous literature reports. These results are similar to recently published real-world PH rates and mortality rates in-stent retriever registries (33). There was no significant difference in the incidence of sICH, PH, and mortality between the RS group and the non-RS group in this study. However, the high rate of sICH in our study might be partly explained by the different populations, treatment strategy, and definition of sICH. In the studies of Asian populations, a similarly high rate of sICH has been found in some studies of Asian populations [Chang et al. (21) 16.7% and Feng et al. (23) 13.6%]. Moreover, all the RS patients used tirofiban, which might be associated with a higher rate of sICH.

At present, there is no consensus on a standard antiplatelet therapy for emergency intracranial stent implantation (20–25), and it is unclear which antiplatelet therapy provides the best balance between HT risk and stent occlusion. Tirofiban has previously been reported to be associated with fatal sICH and adverse clinical outcomes (34); however, in recent years, intravenous tirofiban maintenance for 12–24 h and subsequent treatment with double antibodies have been widely used (6, 7, 20–25), and no significant increase in sICH has been observed. In this study, there was no significant difference in the incidence of sICH between the two groups. This suggests that RS, tirofiban, and subsequent double antiplatelet therapy do not increase the sICH rates. Because of the application of tirofiban and subsequent double antiplatelet therapy, none of the patients who underwent emergency stent implantation had acute or subacute stent thrombosis in this study. Chang et al. (21) believed that glycoprotein IIb/IIIa inhibitors, such as tirofiban, are significantly associated with stent patency and that sICH is not related to any form of antithrombotic aggregation. RS is independently associated with good prognosis without increasing sICH or mortality rates. Moreover, Strackecp analyzed 210 patients who underwent RS in seven neurovascular centers and concluded that the lack of recanalization was an independent predictor of sICH (24).

Before the MT era, studies reported that for AIS with ICAS occlusion, direct stent placement could be used to achieve vascular recanalization, shorten the recanalization time during operation, and reduce the vascular endothelial injury caused by repeated mechanical thrombectomy (35). However, the research of SAMMPRIS and VISSIT (36) showed that the best medical treatment had superior efficacy than that of direct intracranial stent implantation in AIS. Therefore, for patients with AIS with LVO, direct stent placement is not recommended. Remedial measures such as stenting should be considered only after MT fails. In fact, many patients with ICAS LVO can maintain vascular patency through MT, thereby avoiding permanent stent implantation. For example, 86 of 127 patients (67.7%) in this study underwent MT and achieved successful recanalization. Therefore, to treat AIS with ICAS LVO, MT should be carried out first. If recanalization cannot be maintained, further remedial measures, such as stent implantation, should be taken (37).

This study had some limitations. First, the study is conducted with a small sample size and retrospective design, which might have the potential of selection bias. Second, patients in the group with <70% stenosis and insufficient distal flow were not explored in this study due to the small number of cases. This group of patients deserves further exploration. Third, the antiplatelet therapy program in this study was based on the long-term treatment experience and recent research progress at our hospital stroke center. We did not perform platelet function test. This may lead to the deviations in the clinical results.

## Conclusions

In conclusion, AIS with LVO related to ICAS is a common cause of MT failure. RS after MT failure improves the clinical prognosis of patients with acute intracranial atherosclerotic occlusion without increasing the sICH and mortality rates. RS after MT failure might to be a feasible rescue method for treating acute intracranial atherosclerotic occlusion, and it should be considered rather than leaving the patient with a non-recanalized vessel. However, RS is only intended to be a remedial measure for MT failure and should not be used as a conventional approach. In addition, more research is needed to optimize the timing of RS and antiplatelet therapy regimen. Although an increasing number of studies on RS have been published, most of them are retrospective studies, and prospective studies are needed to determine the safety and efficacy of RS.

## Data availability statement

The raw data supporting the conclusions of this article will be made available by the authors, without undue reservation.

## Ethics statement

The studies involving human participants were reviewed and approved by the Human Ethics Committee of Zhejiang Provincial People's Hospital. The patients/participants provided their written informed consent to participate in this study. Written informed consent was obtained from the individual(s) for the publication of any potentially identifiable images or data included in this article.

## Author contributions

SG: drafting the manuscript, data collection for the whole trial, data analysis, and interpretation of data. TJ: drafting the manuscript, data collection and cleaned the data, and revising the manuscript. CX: wrote the statistical analysis plan and revising the manuscript. WH: data collection and cleaned the data. ZS: data analysis and interpretation of data. YG: study concept, study supervision, interpretation of data, revising the manuscript critically for intellectual content, and final approval of the version to be published. All authors agree to be accountable for all aspects of the work in ensuring that questions related to the accuracy or integrity of any part of the work are appropriately investigated and resolved.
